# Secondhand smoke is positively associated with pre-frailty and frailty in non-smoking older adults

**DOI:** 10.3389/fpsyt.2022.1095254

**Published:** 2022-12-15

**Authors:** Zhenmei Fu, Tian Zhou, Fanghong Dong, Mengchi Li, Xuechun Lin, Weixia Ma, Yuting Song, Song Ge

**Affiliations:** ^1^Department of Radiology, Shandong Provincial Hospital Affiliated to Shandong First Medical University, Jinan, Shandong, China; ^2^School of Nursing, Xuzhou Medical University, Xuzhou, Jiangsu, China; ^3^School of Nursing, Hebei University, Baoding, China; ^4^Johns Hopkins University School of Nursing, Baltimore, MD, United States; ^5^Department of Nutrition and Food Hygiene, Hubei Key Laboratory of Food Nutrition and Safety, Ministry of Education Key Laboratory of Environment and Health, School of Public Health, Tongji Medical College, Huazhong University of Science and Technology, Wuhan, China; ^6^Department of Pulmonary and Critical Care Medicine, Shandong Provincial Hospital Affiliated to Shandong First Medical University, Jinan, Shandong, China; ^7^Qingdao University School of Nursing, Qingdao, Shandong, China; ^8^Department of Natural Sciences, University of Houston-Downtown, Houston, TX, United States

**Keywords:** cotinine, cognitive function, older adults, NHANES, secondhand smoke, tobacco

## Abstract

**Introduction:**

Either exposure to secondhand smoke (SHS) or frailty has been linked to adverse health outcomes in nonsmoking adults. However, their relationship is rarely studied. The purpose of this study is to examine the association between serum cotinine level and frailty status among non-smoking older adults.

**Method:**

The study population consisted of 2,703 older adults aged ≥60 from the National Health and Nutrition Examination Survey 2011–2014. Non-smokers were included based on (1) a serum cotinine level ≤ 10 ng/mL and 2) a response of “no” to the question, “Do you currently smoke?” Frailty status was measured based on the Fried Phenotype and had three groups- robust, pre-frailty, and frailty. Multinomial logistic regression models were constructed to examine the association between serum cotinine level quartile and frailty status, controlling for age, sex, race/ethnicity, education, depressive symptoms, alcohol use, and systolic blood pressure.

**Results:**

About half of the participants (median age 70.0 years, range 64–78) were female (53.6%), non-Hispanic White (48.3%), and completed some college and above (50.1%). Multinomial logistic regression with a reference group being those in the 1^st^ quantile (the lowest) of serum cotinine level showed that participants in the 4^th^ quartile (the highest) of serum cotinine level had increased odds of pre-frailty vs. robust (OR 1.522, 95% confidence interval [CI] 1.060, 2.185, *P* = 0.023) as well as increased odds of frailty vs. robust (OR 2.349, 95% CI 1.081, 5.107, *P* = 0.031).

**Conclusions:**

Higher serum cotinine level is associated with increased risk of pre-frailty and frailty versus robust in non-smoking older adults. Prevention and reduction of SHS in older adults may help protect them from developing pre-frailty or frailty.

## Introduction

Frailty and pre-frailty is a prominent aging-related symptom and is very common in older adults (1). Frailty is caused by the lifetime cumulative degradation in multiple physiological systems and is characterized by poor resolution of homeostasis following an external stressor (2). Pre-frailty is a condition predisposing a person to frailty and usually preceding the onset of frailty. According to a systematic review, the overall weighted prevalence of frailty in community-dwelling older adults aged 65 and older was 10.7%, ranging widely from 4.0 to 59.1% in various communities (1). Frailty is frequently referred to as a stage in between successful aging and disability (3). With frailty, the physical, mental, and social engagement of older persons are significantly compromised along with a number of other health indicators (4). As a result, older adults with frailty have an increased risk of falling, disability, immobility, hospitalizations, and lower quality of life (3). Early detection and intervention are crucial to avoid or reverse the development of frailty and its detrimental effects because frailty is a dynamic process and can be seen as a controllable state, especially in the early stages (5–7).

Another prevalent health threat in older adults is exposure to secondhand smoke (SHS). SHS refers to situations when a nonsmoker is exposed to either side-stream or mainline smoke and thus inhales other's smoke (8). SHS is common in older adults (9) and exposure to SHS causes an estimated 41,000 deaths each year among adults in the United States (10). Older adults are particularly vulnerable to the effects of tobacco exposure due to physiologic changes associated with aging and complex medical conditions (11, 12). The associations between active smoking and frailty has suggested smoking as a predictor of worsening frailty status in community-dwelling populations (13). Although many studies have examined the relationship between active smoking and frailty (13), the effect of SHS on frailty is poorly studied (11). To the best of our knowledge, there is only one published study on this topic (11). Therefore, a research gap exists on the impact of SHS on frailty in older adults. In this study, we examined the association between serum cotinine level, a biomarker of tobacco exposure, and frailty status in a group of non-smoking older adults using the National Health and Nutrition Examination Study (NHANES) from 2011 to 2014. The results of this study will provide implications for clinical practice and policy development aimed at preventing frailty among the growing number of older adults.

## Methods

### The parent study design

The NHANES is a continuous cross-sectional survey of civilian, non-institutionalized, America-residing adults and children led by the National Center for Health Statistics of the Centers for Disease Control and Prevention (CDC) (45). Participants with wide sociodemographic regions in the US are recruited using a complex, multistage probability strategy every 2 years (14). Participants' sociodemographic, health, and nutritional status are evaluated using in-person home interviews as well as health exams conducted at mobile exam centers.

For this analysis, the NHANES 2011-2012 (*n* = 9,338) and 2013–2014 (*n* = 9,813) were merged. People who (1) aged ≥ 60, (2) were not actively smokers at the time of the survey, and (3) had no missing information on serum cotinine level and frailty were included. We excluded people (1) who aged <60 (*n* = 15,679), (2) responded “yes” to the question, “Do you currently smoke?” (*n* = 444), (3) had a serum cotinine level >10 ng/mL (*n* = 325), or had missing values on serum cotinine level or frailty status (*n* = 0). People with serum cotinine levels >10 ng/mL were eliminated from the analysis since this level is almost always present in active smokers (15). Finally, the study population consisted of 2,703 non-smoking older adults aged 60 and above.

### Ethical considerations

The NHANES has received ethical permission from the National Center for Health Statistics Research Ethics Review Board. Because we solely used de-identified, publicly accessible data, the University of Houston-Downtown Committee for the Protection of Human Subjects gave this study an exemption.

### Measures

#### Independent variable: Serum cotinine level quartile (1^st^, 2^nd^, 3^rd^, and 4^th^ quartile)

As the most commonly used biomarker for smoking, cotinine is the main metabolite of nicotine, with a half-life of 15 to 20 h. Serum or urinary cotinine level may be used to detect both active smoking and SHS (16). Serum samples from participants were collected during physical exams, aliquoted, and stored at−20°C until being analyzed. Serum cotinine level was measured using the isotope-dilution high-performance liquid chromatography/atmospheric pressure chemical ionization tandem mass spectrometric (ID HPLC-APCI MS/MS) method. The thorough procedure has been published in another study (17). A blank and two quality-control pools were used in each analytical run. Strict quality control and quality assurance were conducted by the Division of Laboratory Sciences, National Center for Environmental Health, CDC (18). This method for measuring serum cotinine has a lower detection limit (LLOD) of 0.015 ng/mL using the variation from the repeated analysis of a 0.2 ml spiked serum sample.

#### Dependent variable: Frailty status (robust, pre-frailty, or frailty)

Frailty status was classified in accordance with a previous NHANES-based study (19). Based on the Fried Phenotype (20), when three or more of the following conditions were met, the person was determined to be frail, including unintended weight loss, sluggish walking, weakness, fatigue, and a lack of physical activity. One or two of the conditions must be present for pre-frailty to exist, but none of the criteria must be present for robustness. Our definition followed the Fried Phenotype's five frailty dimensions; however, we modified the standards for use based on the NHANES data, which is consistent with a previously published study (19). All the following variables were operated as binary variables (yes or no).

1. Unintentional weight loss was determined by participants' responses to three questions: (a) “How much do you weigh without clothes or shoes?” (b) “How much did you weigh a year ago?” and (c) “was the change between your current weight and weight a year ago intentional?” Low body weight for height was defined as having a body mass index (BMI) less or equal to 22.5 kg/m^2^ or at least 5% unintentional weight loss (responding that their weight loss was not intentional) over the previous year. Other published studies have used the same criteria for determining unintentional weight loss (21, 22).
2. Sluggish walking. With no direct information on walking speed in NHANES, we used similar information to assess whether participants had slow walking speed. When asked, “By yourself and without using any special equipment, how much difficulty do you have walking from one room to another on the same level?” Participants who responded, “with some difficulty,” “with significant difficulty,” or “unable to do” were categorized as having slow walking speed.
3. Weakness. If participants responded, “with some difficulty,” “great difficulty,” or “unable to do” to the question “by yourself and without using any special equipment, how much difficulty do you have lifting or carrying something as heavy as 10 pounds?” they would be categorized as having weakness.
4. Fatigue. If participants responded, “with some difficulty,” “great difficulty,” or “unable to do” to the question “by yourself and without using any special equipment, how much difficulty do you have walking for a quarter of a mile?” they would be categorized as having exhaustion.
5. Lack of physical activity. Participants reported an average amount of vigorous and moderately intense activity in minutes for three kinds of activities- work, going to and from locations, and recreation. After that, metabolic equivalent (MET) minutes were computed. One MET minute is equal to 3.5 ml of O2 per kilogram of body weight multiplied by the length of time in minutes spent sitting at rest. It indicates the amount of oxygen consumed while sitting at rest (23). Participants would be categorized as having low physical activity if their MET minutes per week were <600.

#### Covariates

To control for potential confounding between serum cotinine level and frailty status, the following covariates were included in the study- age (years), sex (male or female), race/ethnicity (Mexican Americans, other Hispanics, non-Hispanic White, or non-Hispanic Black), education (below high school, high school graduate, or some college or above), alcohol use (0–1 drink per day, two drinks per day, or three and more drinks per day), depressive symptoms, total cholesterol (mg/dL), and systolic blood pressure (mmHg). All the above information was collected *via* face-to-face interviews or health exams. The Patient Health Questionnaire (PHQ-9) total score was used to represent depressive symptoms (24). The PHQ-9 has shown good reliability and validity among the general population (25).

### Statistical analysis

For descriptive statistics, means (standard deviation) were used to describe continuous data with normal distribution and medians (interquartile range) for continuous data not following a normal distribution. Frequency (percentages) was used to describe categorical data. Multinomial logistic regression models were used to examine the independent relationship between serum cotinine level quartile (reference: 1^st^ quartile, the lowest) and frailty (pre-frailty, frailty, or robust) (reference: robust), controlling the covariates mentioned above. Prior to constructing the regression model, we examined whether multicollinearity existed among the covariates with the resulting variance inflation factor (VIF) less than ten, indicating no multicollinearity (26). A 95% confidence interval (CI) (11) excluding one or a *P*-value < 0.05 was considered as statistical significance. All analyses were performed using SPSS 25.0.

## Results

The characteristics of the study population were presented in Table 1. The 2,703 participants had a median age of 70.0 years, ranging from 64 to 78. About half of them were female (53.6%), non-Hispanic White (48.3%), completed some college or above (50.2%), had a BMI ≥ 25 kg/m2 (73.4%), had 0–1 drink of alcohol use per day (29.3%), and were never smokers (58.2%). The participants had a median of 188.0 mg/dL total cholesterol (range 161.0, 217.0) and 131.3 mmHg systolic blood pressure (range 120.0, 143.3). Their mean serum cotinine level (ng/mL) was 0.13 (SD 0.60), ranging from 0.01 to 9.90. In terms of their frailty status, most of them had pre-frailty (41.7%), followed by robust (29.7%), and frailty (6.4%).

**Table 1:** Characteristics of the participants by serum cotinine level quartile.

**Variables**	**Quartile 1 ≤0.01 ng/ml** **(*n* = 1,203)**	**Quartile 2** **0.01 < cotinine ≤ 0.02 ng/ml** **(*n* = 451)**	**Quartile 3** **0.02 < cotinine ≤ 0.04 ng/ml (*n* = 398)**	**Quartile 4** **>0.04 ng/ml** **(*n* = 651)**	**Total** **(*n* = 2,703)**
Age, years	71.0 (65.0, 79.0)	69.0 (64.0, 78.0)	69.0 (63.0, 75.3)	68.0 (63.0, 76.0)	70.0 (64.0, 78.0)
**Sex**, ***n*** **(%)**					
Male	516 (42.9%)	216 (47.9%)	189 (47.5%)	332 (51.0%)	1,253 (46.4%)
Female	687 (57.1%)	235 (52.1%)	209 (52.5%)	319 (49.0%)	1,450 (53.6%)
**Race/ethnicity**, ***n*** **(%)**					
Mexican Americans	123 (10.2%)	49 (10.9%)	28 (7.0%)	49 (7.5%)	249 (9.2%)
Other Hispanics	123 (10.2%)	55 (12.2%)	50 (12.6%)	43 (6.6%)	271 (10.0%)
Non-Hispanic Whites	717 (59.6%)	182 (40.4%)	163 (41.0%)	243 (37.3%)	1,305 (48.3%)
Non-Hispanic Blacks	148 (12.3%)	98 (21.7%)	87 (21.9%)	225 (34.6%)	558 (20.6%)
Other	92 (7.6%)	67 (14.9%)	70 (17.6%)	91 (14.0%)	320 (11.8%)
**Education**, ***n*** **(%)**					
Below high school	256 (21.3%)	133 (29.5%)	112 (28.2%)	222 (34.2%)	723 (26.7%)
High school graduate	247 (20.5%)	96 (21.3%)	106 (26.7%)	172 (26.5%)	621 (23.0%)
Some college or above	699 (58.2%)	222 (49.2%)	179 (45.1%)	255 (39.3%)	1,355 (50.1%)
**Alcoholic drinks/day**, ***n*** **(%)**					
0–1 drink	393 (63.8%)	144 (65.2%)	116 (60.1%)	140 (46.4%)	793 (29.3%)
2 drinks	164 (26.6%)	47 (21.3%)	45 (23.3%)	83 (27.5%)	339 (12.5%)
3 or more drinks	59 (9.6%)	30 (13.6%)	32 (16.6%)	79 (26.2%)	200 (7.4%)
Depressive symptoms	2.0 (0.0, 4.0)	1.0 (0.0, 4.0)	2.0 (0.0, 5.0)	1.0 (0.0, 5.0)	1.0 (0.0, 4.0)
Total cholesterol, mg/dL	190.0 (162.0, 218.0)	185.0 (156.0, 218.0)	188.0 (159.0, 218.5)	186.0 (161.0, 213.0)	188.0 (161.0, 217.0)
Systolic blood pressure, mmHg	131.3 (120.0, 143.3)	132.0 (119.3, 142.7)	129.3 (117.8, 142.0)	131.3 (120.7, 144.7)	131.3 (120.0, 143.3)
**Frailty**, ***n*** **(%)**					
Robust	369 (38.3%)	142 (39.3%)	138 (45.0%)	155 (32.8%)	804 (29.7%)
Pre-frail	524 (54.4%)	194 (53.7%)	148 (48.2%)	262 (55.5%)	1,128 (41.7%)
Frail	71 (7.4%)	25 (6.9%)	21 (6.8%)	55 (11.7%)	172 (6.4%)

Data was presented as median (interquartile range) for continuous variables and n (%) for categorical variables.

Multinomial logistic regression (Tables 2, 3) with a reference group being those in the 1^st^ quartile (the lowest) of serum cotinine level showed that participants in the 4^th^ quartile (the highest) of serum cotinine level had increased odds of pre-frailty vs. robust (OR 1.522, 95% CI 1.060, 2.185, *P* = 0.023) as well as increased odds of frailty vs. robust (OR 2.349, 95% CI 1.081, 5.107, *P* = 0.031). No significance was found in pre-frailty or frailty for the 2^nd^ and 3^rd^ quartiles, compared with the 1^st^ quartile.

**Table 2:** The independent associations of serum cotinine level quartile (reference: ≤0.01 ng/ml) with pre-frailty.

**Model, pre-frail vs. robust**			**OR (95% CI)**	***P*-value**
Independent variable	Serum cotinine level, ng/ml	Quartile 4 >0.04	1.522 (1.060, 2.185)	**0.023**
		Quartile 3 0.02 < cotinine ≤ 0.04	0.780 (0.528, 1.151)	0.211
		Quartile 2 0.01 < cotinine ≤ 0.02	0.942 (0.656, 1.354)	0.748
		Quartile 1 ≤0.01	Reference	*-*
Control variables	Age, years		1.038 (1.016, 1.061)	**0.001**
	Depressive symptoms		1.101 (1.055, 1.150)	**<0.001**
	Total cholesterol, mg/dL		0.998 (0.995, 1.002)	0.331
	Systolic blood pressure, mmHg		0.997 (0.990, 1.005)	0.465
	Sex	Male	0.524 (0.394, 0.698)	**<0.001**
		Female	Reference	*-*
	Race/ethnicity	Mexican Americans	0.747 (0.399, 1.397)	0.361
		Other Hispanics	0.549 (0.300, 1.007)	0.053
		Non-Hispanic Whites	0.528 (0.332, 0.839)	**0.007**
		Non-Hispanic Blacks	0.540 (0.318, 0.919)	**0.023**
		Other	Reference	*-*
	Education	Below high school	1.676 (1.116, 2.518)	**0.013**
		High school graduate	1.180 (0.849, 1.639)	0.325
		Some college or above	Reference	*-*
	Alcoholic drinks/day	0–1 drink	0.949 (0.629, 1.430)	0.801
		2 drinks	1.097 (0.714, 1.687)	0.673
		3 or more drinks	Reference	*-*

Bolded values mean statistical significance (*P* < 0.05).

**Table 3:** The independent associations of serum cotinine level quartile (reference: ≤0.01 ng/ml) with frailty.

**Model, frail vs. robust**			**OR (95% CI)**	***P*-value**
Independent variable	Serum cotinine level, ng/ml	Quartile 4 >0.04	2.349 (1.081, 5.107)	**0.031**
		Quartile 3 0.02 < cotinine ≤ 0.04	0.714 (0.265, 1.923)	0.506
		Quartile 2 0.01 < cotinine ≤ 0.02	0.790 (0.313, 1.996)	0.618
		Quartile 1 ≤0.01	Reference	*-*
Control variables	Age, years		1.087 (1.033, 1.144)	**0.001**
	Depressive symptoms		1.229 (1.151, 1.313)	**<0.001**
	Total cholesterol, mg/dL		0.994 (0.986, 1.002)	0.167
	Systolic blood pressure, mmHg		0.996 (0.978, 1.013)	0.622
	Sex	Male	0.248 (0.121, 0.508)	**<0.001**
		Female	Reference	*-*
	Race/ethnicity	Mexican Americans	1.107 (0.207, 5.924)	0.906
		Other Hispanics	0.553 (0.104, 2.931)	0.486
		Non-Hispanic Whites	0.942 (0.253, 3.502)	0.929
		Non-Hispanic Blacks	1.236 (0.301, 5.065)	0.769
		Other	Reference	*-*
	Education	Below high school	3.489 (1.553, 7.840)	**0.002**
		High school graduate	1.142 (0.523, 2.494)	0.739
		Some college or above	Reference	*-*
	Alcoholic drinks/day	0–1 drink	1.173 (0.428, 3.212)	0.756
		2 drinks	0.857 (0.281, 2.617)	0.787
		3 or more drinks	Reference	*-*

Bolded values mean statistical significance (*P* < 0.05).

## Discussion

In this group of 2,703 non-smoking older adults, we found that almost half of them had either pre-frailty or frailty. Higher serum cotinine level (>0.04 ng/ml) is associated with an increased risk of pre-frailty and frailty vs. robust. Although our results need to be validated by future prospective studies, they indicate that prevention and reduction of SHS in older adults may prevent them from developing pre-frailty or frailty. Our findings provided implications for clinical practice and policy development aiming at preventing frailty among the growing number of older adults.

Studies have identified positive associations between SHS and cardiovascular diseases (27), stroke (28), lung cancer (29–32), diabetes (33), and all-cause mortality (34) among adults. In addition, a systematic review has found that active smoking predicts the worsening frailty status in various community-dwelling populations (13). However, to date, extremely limited research has been conducted to illustrate the association between SHS and frailty in older adults. To our knowledge, there is only one published study that examined the relationship between SHS and frailty using the NHANES III data collected from 1988 to 1994 (11). Similar to our findings, García-Esquinas et al. (11) found that ~6% of older adults aged 60 years and older had frailty and those in the 4^th^ quartile of serum cotinine, compared with those in the lowest quartile of serum cotinine, were more likely to be frail (OR 2.51, 95% CI 1.06, 5.95). In addition to the findings similar to those of García-Esquinas et al. (11) our study provided additional evidence on the association between SHS and pre-frailty in older adults. In another study (*n* = 71), Teixeira-Gomes et al. (35) found a statistically higher prevalence of second-hand smokers in the pre-frailty group compared with that in the robust group; however, the relationship was insignificant for the frailty group. The findings of our study clarified the inconsistent findings of that study and addressed the knowledge gap on SHS and frailty pointed out by Teixeira-Gomes et al. (35). In another study on SHS, although researchers did not directly assess frailty, they examined grip strength and concluded that even low levels of exposure to SHS were associated with decreased grip strength (36).

The possible mechanism that explains the relationship between SHS and pre-frailty status is unclear but may be multifactorial. Tobacco smoke contains a mixture of multiple toxic chemicals and compounds and may impair almost every organ in the human body. Tobacco smoke has been linked to a variety of physical and mental illnesses (37), which may lead to the development of frailty. Specifically, the most prevalent explanation is that the toxic chemicals in tobacco smoke are associated with an increase in inflammatory markers (38). Chronic inflammation may lead to muscle wasting (39), weight loss, weakness, exhaustion, or slow gait, which are the main components of frailty (20). For example, elevated levels of interleukin-6, C-reactive protein, and tumor necrosis factor-α have been shown to decrease muscle mass, muscle strength, as well as other declined physical abilities (40). Research studies also have supported the positive association between interleukin-6 and C-reactive protein and prevalence and incidence of frailty (41, 42). Despite these findings, future studies are expected to clarify the mechanism.

This study has many strengths. Since there is only one study that examined the relationship between SHS and frailty status in non-smoking older adults, our study makes a novel contribution to the literature and fills in a research gap by providing solid evidence of the negative effect of SHS on frailty status in older adults. Our study has strong generalizability because of the size and representativeness of our study population. To ensure that the study population consisted of non-smokers, two criteria were applied to weed out active smokers. Several sociodemographic, lifestyle, mental health, and physical health factors were adjusted to reduce the risk of residual confounding. According to studies, when exposed to tobacco, older persons are more prone to develop diabetes, osteoporosis, cardiovascular disease, chronic renal disease, and respiratory problems with a poorer prognosis compared with the younger group (43). Thus, our study focused on a neglected problem in an at-risk population.

At the same time, several limitations are contained in this study. To start, because of the cross-sectional nature of this study, we are unable to determine the temporal connection between SHS and frailty status. Furthermore, cotinine is only a reflection of a person's recent exposure to tobacco and does not account for their long-term exposure (half-life 15–20 h). In addition, some excluded people with serum cotinine levels> 10 ng/mL may be non-smokers that are heavily exposed to SHS. Future studies are needed to examine the longitudinal relationship between other biomarkers of tobacco exposure that have a longer half-life, such as 4-(Methylnitrosamino)-1-(3-pyridyl)-1-butanol (NNAL) (44) and frailty in non-smoking older adults, especially those residing in developing countries. The mechanisms explaining the relationship between the two variables should also be explored.

The following are the study's clinical implications: Serum cotinine level, a biomarker of tobacco exposure, was found to be a risk factor for both pre-frailty and frailty in non-smoking older adults. To educate the public on the harmful effects of SHS, officials should advocate smoking-free policies and use social media and other educational strategies to help minimize older adults' SHS exposure at home and in public settings. In clinical settings, clinicians should also talk about specific measures to minimize SHS exposure for their older patients. These approaches may help prevent older adults from developing pre-frailty and frailty.

## Data availability statement

The datasets presented in this study can be found in online repositories. The names of the repository/repositories and accession number(s) can be found below: https://www.cdc.gov/nchs/nhanes/index.htm.

## Ethics statement

The NHANES were reviewed and approved by the National Center for Health Statistics Research Ethics Review Board. The participants provided written informed consent to participate in this study.

## Author contributions

ZF, WM, SG, FD, XL, and ML drafted the initial manuscript, designed the study, and searched for literature. TZ and YS conducted statistical analysis. All authors critically revised the manuscript, contributed to the article, and approved the submitted version.
